# A comprehensive database for biological data derived from sewage in five European cities

**DOI:** 10.1093/database/baaf089

**Published:** 2026-01-20

**Authors:** Ágnes Becsei, Patrick Munk, Alessandro Fuschi, Saria Otani, József Stéger, Dávid Visontai, Krisztián Papp, Christian Brinch, Ravi Kant, Ilya Weinstein, Olli Vapalahti, Miranda de Graaf, Claudia M E Schapendonk, Jeroen Roelfsema, Maaike van den Beld, Roan Pijnacker, Eelco Franz, Patricia Alba, Antonio Battisti, Alessandra De Cesare, Valentina Indio, Fulvia Troja, Tarja Sironen, Chiara Oliveri, Frédérique Pasquali, Ivan Liachko, Benjamin Auch, Colman O’Cathail, Krisztián Bányai, Magdolna Makó, Péter Pollner, Marion Koopmans, Istvan Csabai, Daniel Remondini, Frank M Aarestrup

**Affiliations:** Department of Physics of Complex Systems, ELTE Eötvös Loránd University, Pázmány Péter stny. 1A, 1117 Budapest, Hungary; National Food Institute, Technical University of Denmark, Henrik Dams Allé, Bygning 204, 2800 Kgs Lyngby, Denmark; Department of Physics and Astronomy (DIFA), University of Bologna, Viale Berti Pichat 6/2, Bologna 40127, Italy; National Food Institute, Technical University of Denmark, Henrik Dams Allé, Bygning 204, 2800 Kgs Lyngby, Denmark; Department of Physics of Complex Systems, ELTE Eötvös Loránd University, Pázmány Péter stny. 1A, 1117 Budapest, Hungary; Department of Physics of Complex Systems, ELTE Eötvös Loránd University, Pázmány Péter stny. 1A, 1117 Budapest, Hungary; Department of Physics of Complex Systems, ELTE Eötvös Loránd University, Pázmány Péter stny. 1A, 1117 Budapest, Hungary; National Food Institute, Technical University of Denmark, Henrik Dams Allé, Bygning 204, 2800 Kgs Lyngby, Denmark; Department of Virology, Medicum, University of Helsinki, Haartmaninkatu 3, 00290 Helsinki, Finland; Department of Tropical Parasitology, Institute of Maritime and Tropical Medicine, Medical University of Gdansk, 9b Powstania Styczniowego Street, 81-519 Gdynia, Poland; Department of Basic Veterinary Sciences, Faculty of Veterinary Medicine, University of Helsinki, Agnes Sjöbergin katu 2, 00790 Helsinki, Finland; Department of Virology, Medicum, University of Helsinki, Haartmaninkatu 3, 00290 Helsinki, Finland; Department of Basic Veterinary Sciences, Faculty of Veterinary Medicine, University of Helsinki, Agnes Sjöbergin katu 2, 00790 Helsinki, Finland; Department of Virology, Medicum, University of Helsinki, Haartmaninkatu 3, 00290 Helsinki, Finland; Department of Basic Veterinary Sciences, Faculty of Veterinary Medicine, University of Helsinki, Agnes Sjöbergin katu 2, 00790 Helsinki, Finland; Department of Viroscience and Pandemic and Disaster Preparedness Research Centre, ErasmusMC, Wytemaweg 80, 3015CN Rotterdam, The Netherlands; Department of Viroscience and Pandemic and Disaster Preparedness Research Centre, ErasmusMC, Wytemaweg 80, 3015CN Rotterdam, The Netherlands; Centre for Infectious Disease Control, National Institute for Public Health and the Environment, Antonie van Leeuwenhoeklaan 9, 3721 MA Bilthoven, The Netherlands; Centre for Infectious Disease Control, National Institute for Public Health and the Environment, Antonie van Leeuwenhoeklaan 9, 3721 MA Bilthoven, The Netherlands; Centre for Infectious Disease Control, National Institute for Public Health and the Environment, Antonie van Leeuwenhoeklaan 9, 3721 MA Bilthoven, The Netherlands; Centre for Infectious Disease Control, National Institute for Public Health and the Environment, Antonie van Leeuwenhoeklaan 9, 3721 MA Bilthoven, The Netherlands; Department of General Diagnostics, Istituto Zooprofilattico Sperimentale del Lazio e della Toscana, Via Appia Nuova 1411, Rome 00178, Italy; Department of General Diagnostics, Istituto Zooprofilattico Sperimentale del Lazio e della Toscana, Via Appia Nuova 1411, Rome 00178, Italy; Department of Veterinary Medical Sciences, University of Bologna, Via Tolara di Sopra 50, 40064 Ozzano Emilia (BO), Italy; Department of Veterinary Medical Sciences, University of Bologna, Via Tolara di Sopra 50, 40064 Ozzano Emilia (BO), Italy; Department of Veterinary Medical Sciences, University of Bologna, Via Tolara di Sopra 50, 40064 Ozzano Emilia (BO), Italy; Department of Virology, Medicum, University of Helsinki, Haartmaninkatu 3, 00290 Helsinki, Finland; Department of Basic Veterinary Sciences, Faculty of Veterinary Medicine, University of Helsinki, Agnes Sjöbergin katu 2, 00790 Helsinki, Finland; Department of Physics and Astronomy (DIFA), University of Bologna, Viale Berti Pichat 6/2, Bologna 40127, Italy; Department of Agricultural and Food Sciences, University of Bologna, Viale G. Fanin 44, 40127 Bologna, Italy; Phase Genomics, 1617 8th Ave N, Seattle, WA, United States; Phase Genomics, 1617 8th Ave N, Seattle, WA, United States; European Molecular Biology Laboratory, European Bioinformatics Institute, Wellcome Genome Campus, Hinxton, Cambridge CB10 1SD, United Kingdom; Department of Pharmacology and Toxicology, University of Veterinary Medicine, István u. 2, 1078 Budapest, Hungary; Pathogen Discovery Group, HUN-REN Veterinary Medical Research Institute, Hungária krt. 21, 1143 Budapest, Hungary; Fővárosi Csatornázási Művek Zrt., Asztalos Sándor út 4, 1087 Budapest, Hungary; Data-Driven Health Division of National Laboratory for Health Security, Health Services Management Training Centre, Semmelweis University, Kútvölgyi út 2, 1125 Budapest, Hungary; Department of Biological Physics, ELTE Eötvös Loránd University, Pázmány Péter stny. 1A, 1117 Budapest, Hungary; Department of Viroscience and Pandemic and Disaster Preparedness Research Centre, ErasmusMC, Wytemaweg 80, 3015CN Rotterdam, The Netherlands; Department of Physics of Complex Systems, ELTE Eötvös Loránd University, Pázmány Péter stny. 1A, 1117 Budapest, Hungary; Department of Physics and Astronomy (DIFA), University of Bologna, Viale Berti Pichat 6/2, Bologna 40127, Italy; National Food Institute, Technical University of Denmark, Henrik Dams Allé, Bygning 204, 2800 Kgs Lyngby, Denmark

## Abstract

Sewage metagenomics is a powerful tool for proactive pathogen surveillance and understanding microbial community dynamics. To support such efforts, we present a highly curated and accessible longitudinal dataset of 239 sewage samples collected from five European cities. The dataset, processed through metagenomic sequencing, includes rich analytical outputs such as taxonomic profiles, identified antimicrobial resistance genes, assembled contigs with annotated origins, metagenome-assembled genomes with functional gene annotations, and metadata. Given the computational intensity and time required to reproduce such analyses, we share this dataset to promote reuse and advance research. In addition to the metagenomic data, qPCR was used to identify specific pathogens, and Hi-C sequencing was performed on a subset of the samples to strengthen genomic linkage analysis. Central to this resource is a publicly available PostgreSQL database, designed to facilitate efficient exploration and reuse of the data. This comprehensive database allows users to perform targeted queries, subset data, and streamline access to this extensive resource.

## Introduction

Sewage surveillance has been used for monitoring public health for more than four decades, initially focusing on detecting infectious disease threats such as polio [1], and more recently, it was successfully used for surveillance of SARS-CoV-2 [2, 3]. However, these efforts have primarily relied on targeted PCR-based or targeted sequencing methods. As the cost of next-generation sequencing (NGS) has decreased, this technology has become more accessible for routine use [4]. NGS-based metagenomics enables sequencing of entire microbial communities [5] in both clinical [6] and environmental samples [7]. When combined with strategically selected sampling points (e.g. sewage treatment plants) [8], NGS-based surveillance has the potential to provide simultaneous detection of a broad range of pathogens and antimicrobial resistance genes (ARGs), offering a powerful, data-rich complement to traditional diagnostic and surveillance methods.

Antimicrobial resistance (AMR) poses a significant threat to global health, making it essential to understand its epidemiological patterns to predict the emergence and spread of ARGs.

In 2016, we initiated the global sewage project that involved collection and metagenomic sequencing of sewage from various regions around the world. A key focus of our work has been identifying and quantifying ARGs. Our findings reveal major diversity in the quantity of ARGs [9], and statistical analyses suggest that AMR trends are influenced by indicators related to the national health system and level of sanitation [10, 11]. A longitudinal study on AMR in Copenhagen reveals that AMR levels are relatively stable over time [12]. In addition, we have explored the global composition of the bacteriome [8, 13]. We recovered high- and medium-quality bacterial genomes referred to as metagenome-assembled genomes (MAGs) from sewage and developed a workflow for quantification and correlation to investigate their dynamics over time and geography [8]. Sewage metagenomics combined with urban virome surveillance provides a baseline for a catch-all early warning system for emerging pathogens [14]. Finally, sewage metagenomics also proved effective for studying the distribution of human mtDNA haplogroups in sewage, providing insights into human population genetics [15].

Although the raw sequencing data, as well as available contig, bin, and MAG sequences from these projects, are publicly accessible via platforms such as ENA [16] or NCBI SRA [17], utilizing the datasets for other applications would still require expensive bioinformatic computation and duplicate efforts. This highlights the need for more efficient data sharing practices in scientific research. While platforms such as MGnify [18] offer an automated pipeline for the analysis and archiving of microbiome data, they do not aim to organize different types of analytical output in an interconnected, structured way and do not support sharing of one’s own analytical outputs.

Beyond these technical limitations, the continued use of statements of “data availability upon reasonable request” in scientific papers is still in place although deemed inefficient and unacceptable [19]. To address this issue and effectively avoid duplications, it is crucial to adhere to the FAIR principles [20] of findability, accessibility, interoperability, and reusability. This requires not only a commitment to data sharing but also the development of structured, user-friendly, open-source databases that are reliable, robust, and performant.

In this paper, we present a showcase dataset organized into a PostgreSQL database, which includes detailed metagenomic profiles, ARGs, and other microbiome-related data from sewage samples collected across five European cities: Copenhagen, Rotterdam, Budapest, Rome, and Bologna over 9–19 months with variable time frames between the cities (2019–2021). This includes the raw data from shotgun metagenomic sequencing, assembled contigs, reconstructed MAGs, and qPCR (for pathogens) done on 230 samples, as well as Hi-C sequencing and analysis results for 24 samples. Although the dataset contains 239 sewage samples, certain samples from Copenhagen were sequenced two or three times, leading to a total of 278 metagenomic sequencing samples.

Shotgun sequencing is a technique where DNA is randomly fragmented into small pieces, and each fragment is sequenced independently. The main advantage of this method in bacterial metagenomics compared to targeted sequencing methods (e.g. 16S rRNA sequencing) is that shotgun sequencing uses a non-targeted approach to capture representative data from the entire DNA content of a sample [21].

Once the sequencing data is generated, we assemble the reads into contigs—longer, contiguous sequences formed by overlapping fragments [22]. During metagenomic binning, contigs likely originated from the same organism or genome were grouped together. Bins representing complete or near complete genomes are referred to as MAGs [23].

Proximity-ligation techniques like Hi-C are employed to identify interactions between DNA molecules within the same bacterial cell. This process involves crosslinking DNA fragments that are spatially close in three dimensions, followed by digestion and the formation of chimeric junctions. The resulting DNA fragments are then sequenced using shotgun sequencing. Reads from these Hi-C fragments provide information on the connections between non-contiguous DNA sequences from the same cell. These connections are then used in clustering algorithms to determine which DNA fragments originate from each individual cell. This means Hi-C can potentially link plasmids containing ARGs to their host by physically connecting them to the host genome [24].

## Methods

### Data generation

#### DNA and sequencing data

Untreated sewage samples were collected routinely and during the COVID-19 outbreak between 2019 and 2021 from sewage treatment plants in Rome, Bologna, Rotterdam, Budapest, and Copenhagen. Samples were collected every 2 weeks to obtain time-series data from these European cities. DNA was extracted from these samples, and sequencing data were generated. The sequencing data were derived from the study by Becsei and Fuschi et al. [8].

#### qPCR

qPCR for the detection of selected bacterial pathogens and parasitic protozoans was conducted in six multiplex reactions using a total reaction volume of 20 µl with SensiFast (Bioline, GC Biotech, Waddinxveen, Netherlands) mastermix, primer and probe concentrations of 0.5 and 0.25 µM, respectively, except for *G. lamblia* (primers: 0.25 µM, probe: 0.05 µM). The list of primers used is available on Figshare [25]. PCRs were run on a Lightcycler 480 II instrument (Roche Diagnostics, Basel, Switzerland) using 45 cycles of 5 seconds of denaturation at 95°C and 30 s of annealing at 60°C using Phocid herpesvirus as internal control.

#### Reference-based metagenomic data

Trimmed reads of all libraries were mapped using kma [26] (v1.2.8) with paired-end and singleton files as input against the ResFinder [27] database (commit = 3eedbde) and against a custom genomic database, which was constructed as previously described in the work of Osakunor et al. [28].

Settings of KMA allowed mapping only one query sequence per template and with default penalty values. Resulting mapstat files summarizing abundances in each sample were loaded into the database and are available on Figshare (see the ‘Data records’ section).

#### Assembly-based metagenomic data

In our previous work, reads were assembled to contigs, and MAGs were reconstructed for each individual sample and sampling site via a binning of the contigs followed by the selection of high- and medium-quality MAGs and species-level dereplication. Taxonomic classification, quality information, and quantification results for each MAG in 239 samples were obtained from this work. For detailed description of these workflows refer to Becsei and Fuschi et al. [8].

We used the PPR-Meta [29] (v. 1.1) tool to analyze all contigs obtained from assembly workflows. PPR-Meta, a deep learning-based computational tool classifies metagenomic fragments into phages, plasmids, or chromosomal origins.

In our previous study, assembly of reads from individual samples and pooled reads from each sampling site, followed by binning, resulted in 21 708 166 contigs binned into 34 725 bins. Through dereplication of medium- and high-quality genomes, we identified 2332 distinct prokaryotic species among the MAGs. To label and identify relevant genomic features of these MAGs, they were annotated using Prokka [30] (v1.14.6) with default settings.

#### Hi-C sequencing

250 mg from each sewage pellet (12 from Copenhagen and 12 from Rotterdam) was prepared for Hi-C sequencing protocol using the ProxiMeta™ Hi-C Kit Protocol v4.0 (Phase Genomics, Seattle, USA). Briefly, sewage pellets were resuspended in formaldehyde solution to reach a 1% volume/volume concentration for crosslinking. These samples were left at room temperature for 20 min with periodic gentle stirring. To stop the crosslinking, glycine (from the ProxiMeta™ Hi-C Kit) was added to a final concentration of 1% volume/volume, and the samples were again left at room temperature for 20 min with occasional stirring.

A Hi-C library was constructed using ProxiMeta Hi-C Microbiome v4.0 (Phase Genomics, Seattle, USA) following the manufacturer’s instructions, which are based on Hi-C protocol [31]. Cross-linked DNA from each sample was cut using Sau3AI and MlucI enzymes (from the ProxiMeta™ Hi-C Kit) and then proximity-ligated with biotin-tagged nucleotides to form chimeric molecules from different genomic regions that were close together in the original cells. These chimeras were isolated with streptavidin beads and further processed using the ProxiMeta library preparation reagents. The resulting Hi-C metagenomes were sequenced. The ProxiMeta analysis pipeline (Phase Genomics, Seattle, USA) was employed for analysis of the data [24]. We refer to the resulting groups of contigs as genome clusters. These clusters represent potential genomic sequences from the same organism.

## Results

### Summary of the dataset

The metadata provided comprises GPS coordinates, the names of the sewage treatment plants, and for some cities, temperature and pH measurements. The dataset incorporates outputs of bioinformatic analyses, including abundances of ARGs and different taxa obtained through reference-based classification of sequencing reads. We identified 961 distinct ARGs. Most of these gene hits confer resistance to beta-lactam, aminoglycoside, and tetracycline antibiotics, which are also the most well-represented categories in the ResFinder database [27].

Aligning fragments to a genomic database produced hits across all three superkingdoms (Archaea, Bacteria, and Eukaryota) and identified 3431 distinct genera. Metagenomic assemblies are presented in the form of contigs, contig bins, and MAGs, accompanied by relevant quality assessments and annotations. Gene annotations of the dereplicated medium- and high-quality MAGs reveal a maximum of 9233 and a minimum of 346 coding sequences (CDS) per MAG. Among the 2332 MAGs, 56 encode the 23S, 16S, and 5S rRNA genes, along with tRNAs for at least 18 of the 20 possible amino acids, and have completeness >90% and contamination <5%, thus fulfilling the MIMAG criteria [32] for high-quality MAGs.

The taxonomic classification results for the selected MAGs revealed 879 distinct bacterial genera. Analyzing the possible sources of the contigs identified 14 989154 contigs of plasmid origin, 8346 618 of phage origin, and 44 515 839 of bacterial chromosomal origin. The qPCR tests cover results for 10 bacterial species and 6 protozoan species. ProxiMeta analysis yielded 3105 genome clusters.

**Figure 1.**
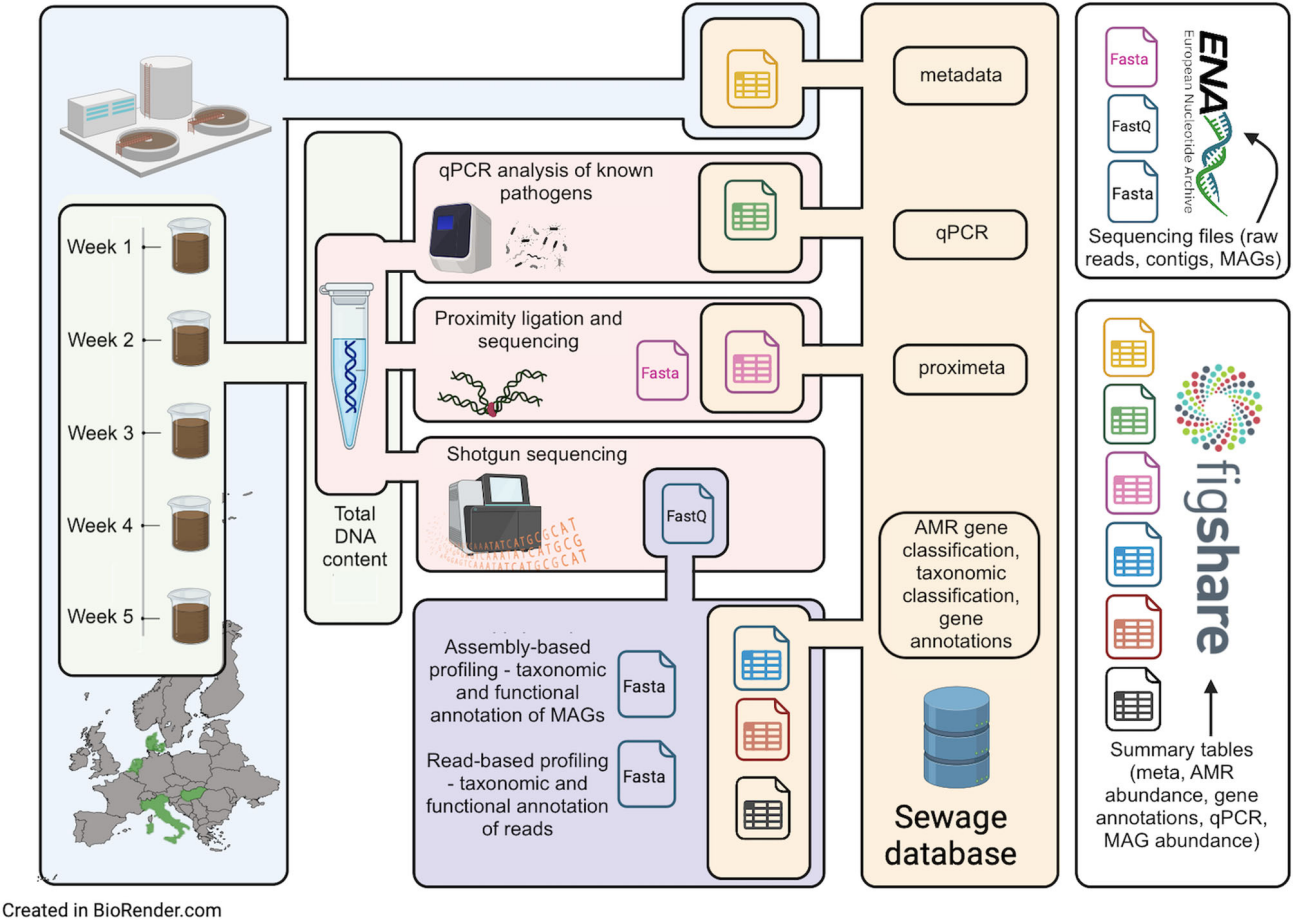
Overview of the dataset. Step 1: Time-series sewage samples were collected from sewage treatment plants across five European cities. Step 2: DNA was extracted from these samples and shotgun sequenced. A subset of samples underwent proximity ligation combined with shotgun sequencing (Hi-C) at Phase Genomics. A specific set of pathogens was quantified with qPCR. Step 3: FastQ files derived from direct shotgun sequencing underwent processing through different metagenomic workflows. These involved mapping trimmed reads against both a genomic sequence database and the Resfinder ARG database, the assembly of reads into contigs, and subsequent binning of these contigs to extract MAGs. Taxonomic identification of MAGs was accomplished using the Genome Taxonomy DataBase (GTDB). Step 4: The outputs from various approaches, along with additional analysis results, were integrated into a PostgreSQL database. Quality-filtered fastQ files and fasta files of contigs, MAGs, and Hi-C clusters were uploaded to the ENA. A subset of tables from the database is available on the Figshare repository (see the ‘Data records’ section).

An overview of the data and how it is organized is presented in Fig. 1.

### Validation of acquired metadata for the samples

All metadata was provided by the involved partners. Metadata had three obligatory entries: sample type, sampling dates and geographical location, which all partners must provide. There were additional entries that partners provided, when possible, e.g. time, temperature and pH. Metadata information was validated with the following: Geographical origin of sample identifiable via openstreetmap.org and sampling date with a specific format: yyyy-mm-dd.

### Validation for MAGs to be accepted in the final dataset

Validation of MAGs is outlined in our previous work [8]. Our goal was to construct a comprehensive, non-redundant, and environmentally representative reference genome dataset covering all sewage samples. A wide array of 34 725 MAGs, originating from two distinct sources: 23 082 genomes from binned contigs of each single sample analysis, and 11 643 genomes from binned contigs of co-assembled samples by site. The primary goal was the selective retention of medium-quality genomes, defined by a contamination level ≤10% and a completeness ≥50%. This selection process yielded a refined collection of 12 687 MAGs. Then we identified and removed duplicate MAGs, culminating in the final collection of 2332 genomes.

### Analytical outputs are stored in a SQL database

**Figure 2.**
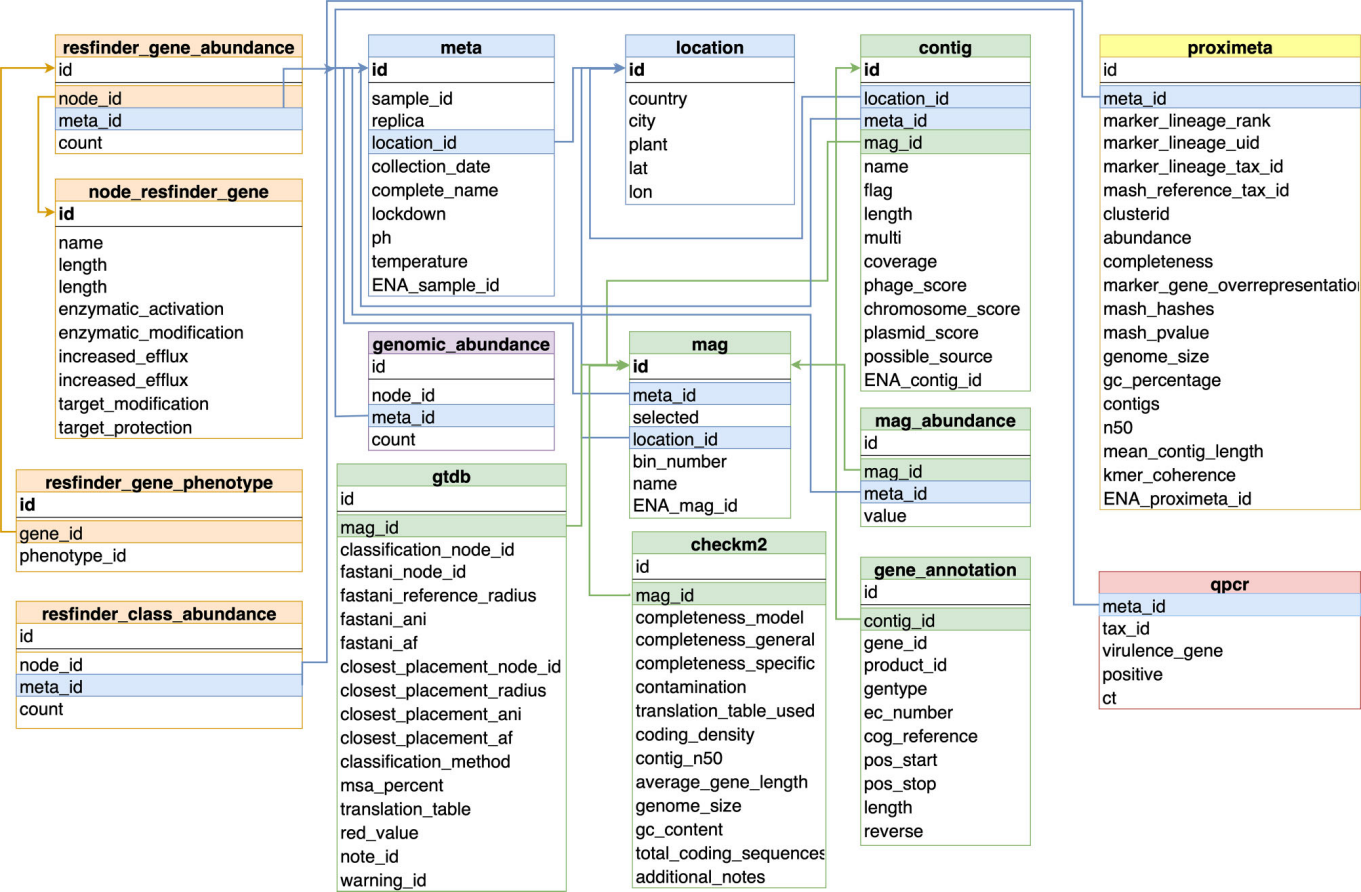
A simplified overview of the database scheme. tables closely related together are coloured according to what kind of information they represent. The ‘meta’ table and the ‘location’ table encompass metainformation. The tables ‘resfinder_gene_abundance’ and ‘resfinder_class_abundance’ joined with detailed gene information (‘node_resfinder_gene’, ‘resfinder_gene_phenotype’) summarize the results of the AMR gene identification. The taxonomic classification of fragments using a genomic database is presented in the ‘genomic_abundance’ table. Results derived from the assembly-based metagenomic workflow include the contig table containing basic contig information and the potential origin of each contig. This ‘contig’ table is linked to the ‘mag’ table providing the association of contigs with their respective MAG, while taxonomy and quality information for MAGs can be found in the ‘gtdb’ and ‘checkm2’ tables. Results from Hi-C sequencing analysis are in table ‘proximeta’. The table ‘qpcr’ stores the results of identification of pathogens through qPCR.

Results in the form of summary Tables of the analyses were organized into a PostgreSQL database referred to as sewage database. During the schema design of the sewage database we balanced between the dogma of canonical data representation and their usability. The simplified diagram of the database is depicted in Fig. 2. The meta information of the samples is stored in two separate Tables to minimize data repetition. These are the ‘meta’ and the ‘location’ Tables. The Table ‘meta’ contains additional information about the samples including collection date, sample type, DNA purification method, IDs for the sample record in the European Nucleotide Archive (ENA) and a reference to the collection site. The ‘replica’ column indicates which sequencing runs were technical replicates of the same sewage sample. The closely related Table, ‘location’, details the sites where samples have been taken, including their country, city, the GPS location information and the name of the plant.

Abundance Tables hold the results of the three different analysis pipelines. Abundance Tables for the ARG classification and genomic reference-based classification approaches (‘resfinder_gene_abundance’, ‘resfinder_class_abundance’, ‘genomic_abundance’) contain the number of reads aligned to each resistance gene or genome. Abundance Table for the high- and medium-quality dereplicated MAG collection called ‘mag_abundance’. This Table contains the number of bases aligned to each MAG per sample.

The results of Hi-C sequencing analysis, including details on the genome clusters, are located in the Table ‘proximeta’, and qPCR results can be found in Table ‘qpcr’.

### Validation of the sewage database

Validation of the database comprises two main components. Technical validation of the database includes ensuring uniformity and consistency in data types, lengths, and formats across corresponding fields in different Tables. Verifying the integrity of primary and foreign keys used for establishing relations between Tables, ensuring referential integrity, and preventing orphaned records. We applied optimization strategies to avoid data redundancy and inconsistencies, particularly when dealing with repeated information across Tables and within a Table. An example includes the introduction of the ‘gtdb_warning’ Table to store each warning message uniquely. Additionally, uniform meta information such as ‘sequencing platform’ shared across all samples was not loaded into the ‘meta’ Table, while making this information available within the database description. We also took advantage of the use of unique constraints the database engine offers to ensure cleanliness of the data. For instance, in Table ‘mag’, it is impossible to store any ad-hoc combination of properties ‘meta_id’, ‘location_id’, and ‘bin_number’, their three-tuple must be unique, which conforms to the definition of the mag. In the database, a version of taxonomy is also represented. There are instances where different taxa share the same name. To uniquely identify a taxon, both the taxon name and its rank (e.g. genus, species, etc.) must be considered together, ensuring that each taxon is distinct.

Under content validation, we conducted a series of diverse queries to ensure that the structural design allows an effective data retrieval. We evaluate the accuracy and validity of the retrieved data from the database in comparison to the original input and expected outcomes. We replicated some of the analysis presented in Becsei and Fuschi et al. [8] study using data retrieved from the database.

## Discussion

This study provides a robust and unprecedented dataset on sewage metagenomics from five European cities, addressing the critical need for comprehensive and accessible data to monitor microbial communities, AMR, and pathogens. By utilizing advanced sequencing techniques such as shotgun metagenomics and Hi-C sequencing, alongside targeted pathogen detection through qPCR, the dataset offers both breadth and depth for microbial surveillance.

The inclusion of 239 samples, processed across diverse workflows, underscores its value as a resource for studying microbial ecology, microbial adaptation, population dynamics, and potential pathogens in urban environments.

A central feature of this study is the development of a PostgreSQL database designed to accommodate and organize the vast array of data generated. This database serves as an accessible and flexible platform that integrates metadata, analytical outputs, and derived genomic features, enabling efficient querying and data exploration. Structured to minimize redundancy and ensure data integrity, it provides a robust foundation for researchers to engage with the data. Tables within the database store essential information, such as sample metadata, ARG abundance, taxonomic profiles, and MAG-related data, which are linked via carefully constructed schema. Furthermore, the inclusion of Hi-C sequencing results and qPCR data in dedicated Tables makes it possible to explore genomic linkages and validate findings across different analytical methods.

Our long-term goal is to support the standardization of organizing and storing metagenomic data. We aim to develop a workflow that facilitates the integration of analytical outputs from various metagenomic pipelines and other connecting data into a unified database structure that can be accessed and re-used by others.

The database’s design also supports future updates, allowing for the inclusion of additional samples or analytical outputs, ensuring its relevance for sewage studies, and expanding applications over time.

Future efforts should also focus on integrating these types of datasets with clinical or epidemiological data [33] to provide a more comprehensive understanding of health-related trends. Additionally, developing user-friendly pipelines or visualization tools tailored to this dataset could enhance accessibility and usability for a broader scientific audience.

In conclusion, this study represents a significant step toward standardized and accessible metagenomic surveillance. The integration of a thoughtfully designed database not only ensures reusability but also sets a benchmark for data sharing and analysis in sewage metagenomics. This concept supports the transition from fragmented spreadsheet based data management to a standardized, queryable platform.

## Data Availability

The data underlying this article are available in the European Nucleotide Archive (https://www.ebi.ac.uk/ena/browser/home) under accession PRJEB68319, and in Figshare, at https://dx.doi.org/10.6084/m9.figshare.25016147.v1.

## Data records

The raw data can be retrieved from the ENA under project number PRJEB68319. The submitted data includes quality-trimmed paired-end files in fastQ format for each library, as well as files containing assembled contig sequences in fasta format for single-sample assemblies and co-assemblies, metagenome assembled genomes, and resulting genome clusters of the Hi-C sequencing analysis in fasta format.

The analytic outputs generated through different metagenomic approaches, qPCR, and Hi-C analysis results, along with the available metadata have been incorporated into a PostgreSQL database and are available for download on the Figshare public data repository [25]

The PostgreSQL database can be accessed through a public web interface. The link to this interface, as well as detailed information and the latest updates regarding the sewage database is available on the following GitHub page: https://github.com/nyeszka/sewage_db.

## Code availability

The software utilized in this study is freely available in the public domain, and the usage parameters are outlined in the ‘Methods’ section. In cases where specific operational parameters were not provided, default parameters were used.
